# Caregiver and adolescent intuitive eating behavior: associations with weight change during family-based treatment for anorexia nervosa

**DOI:** 10.1007/s40519-023-01557-0

**Published:** 2023-03-25

**Authors:** Jillian D. Nelson, Paige J. Trojanowski, Claire M. Aarnio-Peterson, Sarah Fischer, Leah Adams, Abigail Matthews

**Affiliations:** 1 Department of Psychology, George Mason University, 4400 University Drive, Fairfax, VA 22030 USA; 2 Department of Pediatrics, University of Cincinnati College of Medicine, Cincinnati, OH USA; 3 Division of Behavioral Medicine and Clinical Psychology, Cincinnati Children’s Hospital Medical Center, Cincinnati, OH USA

**Keywords:** Intuitive eating, Anorexia nervosa, Family-based treatment, Adolescents

## Abstract

**Purpose:**

Intuitive eating (IE) is an adaptive eating construct for which little research exists in eating disorder (ED) samples. IE is negatively correlated with disordered eating behaviors in healthy adolescents and adults, and similar associations have been found in adults with EDs. This study aims to examine IE in a treatment seeking sample of adolescents and their caregivers to understand the role of IE in weight gain during FBT.

**Methods:**

Descriptive statistics and bivariate correlations were calculated in a sample of 47 pairs of adolescent patients and their caregivers who initiated outpatient FBT at a large academic medical center. Analyses examined associations between caregiver and adolescent IE on the Intuitive Eating Scale (IES), change in percent expected body weight (%EBW) by session 4 and end of treatment (EOT), clinical impairment, and ED pathology.

**Results:**

Significant correlations were found between aspects of adolescent IE, ED symptoms, and clinical impairment. Caregiver IES scores (Reliance on Hunger and Satiety Cues, Body-Food Choice Congruence, IES Total) were negatively related to adolescent ED symptoms (EDE-Q Weight Concerns, EDE-Q Shape Concerns, EDE-Q Global) at baseline. Caregiver IE (Eating for Physical Rather than Emotional Reasons) was positively associated with adolescent weight gain at FBT session 4 and EOT, even when statistically adjusting for gender and initial level of care.

**Conclusion:**

Study results were consistent with past research indicating adolescent IE is negatively associated with ED behaviors, cognitions, and impairment. This study is the first to provide evidence that caregiver IE is positively associated with adolescent weight gain in FBT and is the first to provide evidence that caregiver IE is negatively related to adolescent ED symptoms. Future research should examine adolescent and caregiver IE throughout FBT to understand the role of IE in treatment response.

**Level of evidence:**

Level III: Evidence obtained from cohort or case-control analytic studies.

Eating disorder (ED) research has often focused on the presence of maladaptive eating behaviors and cognitions as opposed to the presence of adaptive ones [1]. Intuitive eating (IE) is an eating construct that is negatively correlated with disordered eating behaviors and cognitions in adults [2] and adolescents [3]. IE is characterized by a strong understanding of and reliance on physiological signals (e.g., hunger/satiety cues) rather than emotional or situational motivators of food intake [1]. Intuitive eaters do not moralize food choices as “good” or “bad” [1]. Although taste is considered, they tend to choose foods that enhance their body functioning and health [1, 4, 5]. One proposed comprehensive definition of ED recovery in women includes IE [6], suggesting the need to further study its’ role in ED treatment.

IE is most commonly measured by the Intuitive Eating Scale (IES-2) [5] which represents IE with four subscales. Intuitive eaters allow themselves to eat when they are hungry, and do not forbid themselves from eating certain foods (i.e., unconditional permission to eat); they eat food when physically hungry and not in response to unwanted emotions (i.e., eating for physical rather than emotional reasons); they trust and rely on internal hunger and satiety cues to guide their eating (i.e., reliance on hunger and satiety cues); and finally, they choose what to eat based on their bodies’ needs (i.e., body-food choice congruence).

## IE in adolescents and young adults

In non-clinical adolescent and young adult samples, IE is associated with fewer ED behaviors, lower body dissatisfaction, and more effective emotional functioning [2, 3, 7]. For example, a large study of middle school boys and girls found that regardless of sex, dieting was associated with feeling less free to eat as pleased (i.e., lower unconditional permission to eat) and with more eating to soothe emotions (i.e., lower eating for physical reasons) [8]. In 12 to 16-year-olds, total IE scores correlated with more body appreciation and less social comparison [9]. In an 8-year longitudinal study, Hazzard et al. [10] found that greater baseline IE in adolescents (*M* age 14.5) and improvements in IE over time were negatively associated with binge eating, unhealthy weight control behaviors (e.g., fasting, skipping meals, vomiting), low self-esteem, and body dissatisfaction at follow-up, suggesting long-term benefits of IE from adolescence into young adulthood [10]. These studies suggest that IE in adolescents and young adults is associated with decreased disordered eating behaviors and cognitions and is positively associated with adaptive body image, self-esteem, and hunger and satiety cues.

## IE in individuals with EDs

Several studies have also investigated IE in clinical ED samples; however, studies have been conducted in almost exclusively adult women. For example, women with a current ED [anorexia nervosa (AN), bulimia nervosa (BN), and binge eating disorder (BED)] had significantly lower IE scores than healthy controls [11].

Other studies have explored the role of IE in ED treatment and recovery. Koller et al. found that women in full recovery had significantly higher IE scores than those in partial recovery or with a current ED, and their scores did not differ from healthy controls [6]. Another study investigated the effects of an IE intervention for women receiving treatment for AN, BN, and other specified feeding or eating disorders (OSFED) in an inpatient treatment center and found that IE scores increased significantly across all diagnoses [12]. At discharge, higher IE scores were associated with better clinical outcomes including fewer ED symptoms, body image concerns, and psychological symptoms [12]. These findings suggest that IE may improve with ED treatment and be associated with better clinical outcomes.

## Parent modeling and IE

Most research examining caregivers’ influence on child eating behavior has been done in parents. Studies suggest that parent feeding and eating practices influence child eating behavior both directly and indirectly through modeling [13, 14]. For example, parental pressure to eat, restriction, modeling, and food availability, have been linked to child food choice and consumption [15]. Parent tendencies to offer food as a form of emotional support are also associated with child emotional eating [16] and contraindicate the IE construct of eating for physical, rather than emotional, reasons. On the other hand, parent conversations that focus on specific eating behaviors and habits instead of conversations about weight loss may protect against disordered eating [17]. Parent comments about child weight/size and parents’ own disordered eating behaviors and cognitions are associated with disordered eating behavior in their youth [17–19]. IE may be modeled by caregivers in the same way as these types of eating behaviors and cognitions. Although research on the association between caregiver IE and child eating behavior is in its infancy, two studies in young children support that maternal unconditional permission to eat, higher trust in internal cues, and eating for physical reasons, as measured by the IES-2, may be protective against restrictive child feeding [20, 21]. Although unconditional permission to eat may be associated with lower self-regulation of food intake and hence weight gain, this again suggests that this construct may be protective against the restriction characteristic of AN.

While research suggests that IE is linked to disordered eating, adolescent and caregiver IE has not been studied in adolescents seeking family-based treatment (FBT) [22]. FBT is an empirically supported treatment with the strongest evidence base for adolescents with AN. Across randomized control trials (RCTs), between 50 and 75% of adolescents in FBT conditions are weight restored, a common marker of recovery, and weight restoration appears stable up to 1 year after terminating treatment [23–25]. In early FBT, weight restoration is prioritized, and caregivers assume control over all eating-related decisions for the adolescent with AN (e.g., planning, preparing, and supervising all meals). Upon achieving a healthy weight, independent eating is gradually returned to the adolescent in a developmentally appropriate fashion.

Secondary data analyses of FBT outcome studies have indicated that early weight gain (in the first 4 weeks of treatment) is a potential marker for later recovery [26, 27]. Not surprisingly, accumulating data also illustrates the influence of parent behaviors on FBT outcomes [28–35]. For example, parent self-efficacy, or a parent’s ability to take charge of their child’s eating and weight-related behaviors, mediates the relationship between FBT and weight restoration [29]. Very little is known about how caregivers’ personal eating attitudes and behaviors impact FBT outcomes. It is conceivable that caregiver IE influences eating-related decisions in FBT and could indirectly impact treatment effectiveness. For example, a caregiver who labels certain foods as forbidden and has difficulty choosing foods that honor energy needs (i.e., demonstrating lower IE) may struggle to provide their adolescent with high-fat, calorically dense foods that may be necessary for weight restoration. Alternatively, caregivers with higher IE may have more success in facilitating their adolescent’s weight gain than caregivers who endorse less adaptive eating attitudes. There is a dearth of research on caregiver eating attitudes and IE and how these may influence FBT, so answers to these questions are unknown.

## Study aims

The current study had four related aims. First, this study aimed to describe baseline adolescent IE and its connection to ED pathology and clinical impairment in an FBT-seeking sample of adolescents with AN or atypical AN (AAN). Second, the relationship between baseline adolescent IE and weight gain during FBT was explored at FBT session 4 and end of treatment (EOT). To control for individual differences in body weight, weight gain was measured as the change in percent expected body weight (%EBW) which is described in the Method section below. Session 4 was selected as a time point of interest given the evidence that early weight gain in FBT is a potential predictor of later recovery [26, 27]. Given the connection between parent and child feeding and eating behaviors, the third aim of the study was to explore the relationship between caregiver IE and adolescent IE at baseline in an FBT-seeking sample. The relation between parent modeling and child eating behavior [e.g., 13, 20, 21], suggest it is possible that caregiver and adolescent IE are positively associated. However, this study includes a clinical sample of adolescents with AN/AAN, and research suggests lower IE in individuals with EDs. Thus, caregiver and adolescent IE at baseline may not be positively associated. Last, the association between baseline caregiver IE and adolescent change in %EBW during FBT was explored at FBT session 4 and EOT. It is possible that caregiver IE is associated with weight gain during FBT, as the responsibility for re-nourishment and weight gain is placed solely on caregivers in this treatment. However, there is a dearth of prior research in this area and all hypotheses are exploratory.

## Method

Participants were identified via retrospective chart review of all adolescents with AN or AAN who received multidisciplinary ED care at a large pediatric medical center in the Midwestern United States between July 2017 and January 2019. Patients were initially evaluated by an adolescent medicine physician within the ED program and if medically stable, were referred for outpatient FBT. Medically unstable patients (e.g., bradycardia, electrolyte imbalance, severe malnutrition and/or high risk for refeeding syndrome), were hospitalized for acute medical stabilization, received an FBT-guided intervention while admitted, and discharged to outpatient FBT [36]. Patients were excluded if they and/or their caregiver did not speak fluent English and if they had prior ED treatment. Forty-seven pairs of adolescents and a primary caregiver were identified, with patients ranging in age from 11 to 18 years (M = 15.14, SD = 1.43). One patient identified as Asian and non-Hispanic while the remaining identified as White and non-Hispanic. Forty-one adolescents identified as cisgender female (87.2%) and 6 as cisgender male (12.8%). Participating caregivers included 34 mothers (72.3%), 5 fathers (10.6%), and one legal guardian (2.4%). Seven caregivers (14.9%) did not report their relationship to the patient.

Patients and caregivers completed a battery of psychosocial assessment questionnaires at treatment baseline, in line with standard clinical care. For outpatients, questionnaires were completed electronically, prior to FBT session 1. For inpatients, electronic questionnaires were completed within 48-h of admission to the hospital. Notably, for all study participants, whether recruited as inpatients or outpatients, all assessments were completed *prior* to beginning FBT. In this chart review, psychosocial questionnaires were only available at treatment baseline, not at EOT or follow up sessions.

Patient weights over the course of FBT were extracted retrospectively via chart review of multidisciplinary clinic visits. Weights were extracted to correspond with three time points, including: (1) psychosocial questionnaire completion (i.e., baseline); (2) FBT session 4; (3) end of treatment. For the purpose of this analysis, change in %EBW was calculated from baseline (i.e., prior to FBT session 1). Importantly, weight gain on the inpatient unit was NOT considered as part of the longitudinal weight gain analysis in this study. Instead, for those participants who received inpatient treatment, weight gain for this analysis was calculated from their discharge weight, representing the point at which parents assumed control over their adolescent’s eating. Notably, for our inpatients, they initiate FBT within 2 weeks of discharge. Retrospective chart review was utilized to extract weights at FBT session 4 and EOT. All participants in our sample completed treatment through FBT session 4. Approximately 75% (n = 35) completed a full course of FBT, and 12 participants in our sample (25.5%) dropped out before EOT. Among those who dropped out, 6 were dissatisfied with the FBT approach and desired individual therapy (50%), 4 were lost to follow-up (i.e., ceased scheduling appointments despite recommendations for ongoing treatment) (33.3%), one was admitted to residential treatment due to lack of progress in FBT (8.3%), and one believed that they were recovered despite recommendations for ongoing weight restoration (8.3%). This study was approved by the Institutional Review Board. Informed consent and assent were provided at baseline assessment, allowing for the inclusion of current and future clinical data in a research database.

### Measures

#### Intuitive eating scale-2 (IES-2) [5]

Adolescents and their caregivers completed the IES-2, a 23-item self-report measure which assesses IE behaviors by calculating a Total score and four subscales scores: Unconditional Permission to Eat (UPE; eating when hungry and not forbidding certain foods), Eating for Physical rather than Emotional reasons (EPR; eating when physically hungry, not to cope with unwanted emotions), Reliance on Hunger and Satiety Cues (RHSC; trusting in and relying on internal hunger or satiety cues to guide eating), and Body-Food Choice Congruence (BFCC; matching food choices to their body’s needs). Each item is scored on a 5-point Likert scale (1 = *“Strongly disagree”* to 5 = *“Strongly agree”*), with higher total and subscale scores reflecting greater presence of IE. The IES-2 has demonstrated reliability and validity in college samples of men and women [5]. In the current study, internal consistency for each subscale ranged from fair to excellent (adolescent UPE α = 0.757, adolescent EPR α = 0.860, adolescent RHSC α = 0.933, adolescent BFCC α = 0.787, caregiver UPE α = 0.773, caregiver EPR α = 0.926, caregiver RHSC α = 0.936, caregiver BFCC α = 0.861) for adolescent and caregiver scores.

#### Eating disorder evaluation-questionnaire (EDE-Q)[37]

Adolescents completed the EDE-Q, a 28-item self-report questionnaire which assesses the frequency or intensity of ED symptoms over the past 28 days [37]. Each item is rated on a 7-point Likert scale (from 0 to 6) with a score of 4 or higher indicating clinical severity. It yields 4 subscale scores (Restraint, Eating Concern, Shape Concern, and Weight Concern) and a Global score. Higher scores indicate greater symptom severity. In the current study, the internal consistency for EDE-Q subscales and the Global scale ranged from good to excellent (Restraint α = 0.86, Eating Concern α = 0.82, Shape Concern α = 0.95, Weight Concern α = 0.81, Global score α = 0.89).

#### Clinical impairment assessment questionnaire (CIA) [38]

Adolescents in our sample completed the CIA, a 16-item self-report measure which assesses the psychosocial impairment experienced by individuals with ED symptoms or behaviors over the past 28 days [38]. Items are rated on a 4-point Likert scale (0 = *“Not at all”* to 3 = *“A lot”*) and summed into a total score, with higher scores indicating greater impairment. A score of 16 is the cut-point for predicting a full-threshold ED [39]. The CIA is a validated measure of psychosocial impairment and has been found to predict treatment outcomes [40]. The internal consistency in the current sample was excellent (α = 0.95).

#### Medical chart review

Demographic and clinical characteristics were obtained through retrospective review of electronic medical records, including patient age, gender identity, race, ethnicity, diagnosis (AN or AAN), length of illness (months), level of care at baseline (inpatient or outpatient), expected BMI percentile for age-and sex (measured by the patient’s median BMI percentile for age-and-sex prior to illness development), and weight and height at baseline (prior to FBT session 1), FBT session 4, and EOT. For outpatients (n = 13, 17.7%), baseline weight and height were obtained prior to FBT session 1. For inpatients (n = 34, 72.3%), hospital discharge weight was used as baseline weight (also prior to FBT session 1), representing the weight at which caregivers assumed control over their adolescent’s eating behaviors.

#### Percent expected body weight (%EBW)

Within the clinical ED program, %EBW is used to assess progress toward weight restoration goals. Notably, EBW is determined by developmental growth trends versus median body weight (MBW), which is determined from the 50th percentile for age-and-sex. Further, over half of our sample (n = 25, 53.2%) had AAN, with premorbid growth trends above the 50th percentile, so use of MBW to establish weight goals would not reflect historical growth patterns. In analyses, baseline EBW was calculated by inputting each patient’s height into their individualized, expected BMI percentile for age-and-sex. Next, %EBW was calculated for each time point of interest (baseline, FBT session 4, EOT) by dividing the patient’s actual weight at each time point by EBW and multiplying by 100.

### Data analysis

Five participants did not have data for the CIA, but there were no item-level missing data in the dataset. Missing data were deleted list-wise for analyses involving the CIA. Descriptive statistics were conducted for demographic variables (age, gender identity, race, ethnicity, diagnosis, length of illness, level of care at baseline, caregiver type) and clinical characteristics (adolescent and caregiver IE, ED symptoms, clinical impairment). Additionally, we compared participant outcomes and baseline variables on attrition and level of care.

Several factors were considered in planning statistical analyses. Longitudinal data was available for weight gain and %EBW, as well as several clinically relevant questionnaires at the baseline assessment. Given that questionnaire data was not available at longitudinal timepoints, we were not able to conduct analyses which modeled how changes in weight impacted changes in IE or vice versa. We were only able to examine how baseline IE was associated with change in weight over time. Next, an a priori power analysis, conducted with G*Power 3.1.9.6 [41], found our sample size lacked sufficient power to examine how all subscales of IE (entered simultaneously) at baseline impacted longitudinal weight gain/%EBW using multiple regression analysis. With a sample size of 42 participants at EOT, we had 80% power to detect a small effect (r = 0.28) using three predictors in the model. Thus, bivariate correlations were used to examine associations between adolescent and caregiver IE (IES-2 subscale and Total scores), ED symptoms (EDE-Q subscale and Global scores), clinical impairment (CIA), and change in %EBW from baseline to FBT session 4 and from baseline to EOT.

## Results

### Sample characteristics

Demographic and clinical characteristics of patients and caregivers in our sample are summarized in Tables 1 and 2.

**Table 1:** Adolescent characteristics at baseline (unless otherwise indicated)

	*M* (*SD*)
Age (in years)	15.1 (1.4)
Body Mass Index (kg/m^2^)	18.9 (2.3)
Illness duration (in months)	11.0 (9.8)
Weight change by session 4 (in pounds)	3.8 (5.4)
Weight change by EOT (in pounds)	13.6 (11.6)
Change in %EBW by session 4	8.7 (1.1)
Change in %EBW by EOT	9.5 (0.9)

	n (%)
Gender identity	
Cisgender male	6 (12.8)
Cisgender female	41 (87.2)
Diagnosis	
AN	22 (46.8)
Atypical AN	25 (53.2)
Level of care at admission	
Inpatient	34 (72.3)
Outpatient	13 (27.7)

*M* mean, *SD* standard deviation, *%EBW* percent expected body weight, *EOT* end of treatment

**Table 2:** Mean scores of key adolescent and caregiver variables at baseline

Measure	*M* (*SD*)
Adolescent IES-2 scores	
UPE	2.33 (0.93)
EPR	4.21 (0.66)
RHSC	2.88 (1.02)
B-FCC	3.92 (0.76)
Total	3.34 (0.57)
Caregiver IES-2 scores	
UPE	3.40 (0.88)
EPR	3.65 (0.94)
RHSC	3.30 (0.91)
B-FCC	3.79 (0.79)
Total	3.51 (0.65)
EDE-Q	
Restraint	3.08 (1.88)
Eating concern	2.23 (1.56)
Shape concern	3.36 (2.03)
Weight concern	2.72 (1.75)
Global score	2.85 (1.68)
CIA total	23.31 (12.50)

*M* mean, *SD* standard deviation, *IES-2* Intuitive Eating Scale, *UPE* unconditional permission to eat, *EPR* eating for physical rather than emotional reasons, *RHSC* reliance on internal hunger/satiety cues, *B-FCC* body-food choice congruence, *EDE-Q* Eating Disorder Examination-Questionnaire, *CIA* clinical impairment assessment

### Attrition and comparisons by diagnosis and level of care

Adolescents who dropped out before EOT were compared to those who remained in treatment on demographic variables (i.e., age, BMI, illness duration) and clinical characteristics (i.e., IES-2, EDE-Q, CIA scores). Results of *t*-tests determined that adolescents who dropped out before EOT were significantly younger (t = 2.16, p < 0.05) and had a significantly higher baseline BMI (t = 2.30, p < 0.05) than those who remained in treatment. No other significant differences were detected.

*T*-tests were also conducted to examine potential differences in clinical characteristics between patients with AN compared to patients with AAN. Importantly, there were no significant differences in IES-2, EDE-Q, or CIA scores between the groups. *T-*scores ranged from − 1.20 to 1.13.

We also examined differences in clinical variables for participants at inpatient versus outpatient level of care at baseline. Inpatients had significantly higher IES-2 scores on the EPR (*t* = 2.38, *p* < 0.05) and B-FCC subscales (*t* = 2.41, *p* < 0.05). There was no difference in increase in %EBW between inpatients and outpatients. No other differences were detected.

### Bivariate correlations

Bivariate correlations between adolescent IE, caregiver IE, ED symptoms, clinical impairment, and longitudinal weight gain (weight change in pounds and change in %EBW) are reported in Table 3. Adolescent IES-2 Total, UPE, and RHSC scores, were significantly negatively related to EDE-Q Global and all subscale scores. CIA was significantly negatively correlated with adolescent IES-2 Total and all subscale scores except for B-FCC. Adolescent IES-2 scores, EDE-Q scores, and CIA were not significantly correlated with change in %EBW at FBT session 4 or EOT. Change in %EBW at FBT session 4 was positively associated with change in %EBW at EOT.

**Table 3:** Bivariate correlations between adolescent and caregiver variables

Variable	1	2	3	4	5	6	7	8	9	10	11	12	13	14	15	16	17	18	19
1. Wt. change by session 4																			
2. Wt. change by EOT	0.648**																		
3. Change in %EBW by session 4	0.977**	0.608**																	
4. Change in %EBW by EOT	0.629**	0.968**	0.631**																
5. Adol UPE	− 0.109	0.110	− 0.103	0.107															
6. Adol EPR	− 0.115	0.027	− 0.168	− 0.030	− 0.157														
7. Adol RHSC	0.107	0.263	0.103	0.282	0.636**	0.203													
8. Adol B-FCC	0.255	0.121	0.204	0.067	− 0.019	0.445**	0.338*												
9. Adol IES-2 Total	0.001	0.204	− 0.028	0.178	0.661**	0.514**	0.887**	0.508**											
10. Caregiver UPE	0.031	0.166	0.037	0.173	0.118	0.124	− 0.102	0.023	0.057										
11. Caregiver EPR	0.292*	0.346*	0.275	0.335*	− 0.103	0.220	− 0.034	0.161	0.057	0.119									
12. Caregiver RHSC	0.025	0.048	0.043	0.084	0.242	0.102	0.215	0.391**	0.315*	0.440**	0.420**								
13. Caregiver B-FCC	0.221	0.204	0.221	0.221	− 0.053	0.238	0.184	0.552**	0.258	0.013	0.458**	0.572**							
14. Caregiver IES-2 Total	0.203	0.277	0.203	0.291	0.070	0.231	0.055	0.322*	0.206	0.579**	0.777**	0.827**	0.606**						
15. Restraint	0.109	0.093	0.110	0.097	− 0.578**	− 0.059	− 0.468**	− 0.158	− 0.520**	− 0.004	− 0.049	− 0.332*	− 0.155	− 0.173					
16. Eating Concern	− 0.036	− 0.019	− 0.037	− 0.035	− 0.410**	− 0.246	− 0.454**	− 0.070	− 0.502**	0.073	− 0.227	− 0.262	− 0.233	− 0.223	0.709**				
17. Shape Concern	− 0.008	− 0.066	0.000	− 0.074	− 0.528**	− 0.172	− 0.547**	− 0.218	− 0.593**	0.023	− 0.148	− 0.439**	− 0.334*	− 0.281	0.811**	0.855*			
18. Weight Concern	0.008	− 0.013	0.003	− 0.035	− 0.453**	− 0.200	− 0.549**	− 0.295*	− 0.587**	− 0.033	− 0.163	− 0.492**	− 0.420**	− 0.342*	0.815**	0.813**	0.922**		
19. EDE-Q Global	0.021	− 0.002	0.023	− 0.013	− 0.534**	− 0.178	− 0.544**	− 0.203	− 0.593**	0.014	− 0.153	− 0.414**	− 0.307*	− 0.274	0.901**	0.899**	0.966**	0.954**	
20. CIA Total	0.056	− 0.074	0.057	− 0.051	− 0.397**	− 0.316*	− 0.544**	− 0.150	− 0.607**	0.061	− 0.007	− 0.247	− 0.211	− 0.107	0.558**	0.797**	0.721**	0.734**	0.754**

*Wt* weight, *EOT* end of treatment, *EDE-Q* Eating Disorder Examination-Questionnaire, *CIA* clinical impairment assessment, *UPE* unconditional permission to eat, *EPR* eating for physical rather than emotional reasons, *RHSC* reliance on internal hunger/satiety cues, *B-FCC* body-food choice congruence, *IES* Intuitive Eating Scale

*p < 0.05, **p < 0.01

Bivariate correlations between caregiver and adolescent IES-2 scores are also reported. Caregiver RHSC scores were significantly positively related to adolescent IES-2 Total scores. Caregiver B-FCC scores and adolescent B-FCC scores were also significantly positively related. Caregiver RHSC and caregiver B-FCC scores were both negatively associated with EDE-Q Weight Concern, Shape Concern, and Global scores. Thus, caregivers’ self-reported ability to eat based on hunger cues and match food to their body’s needs were associated with lower concerns about weight and shape in their adolescents. Finally, caregiver EPR scores were positively associated with change in %EBW at EOT. Thus, among patients who completed treatment, caregiver attitudes towards eating were associated with adolescent weight gain through the course of FBT.

### Hierarchical multiple linear regression analyses

We also conducted a series of hierarchical multiple linear regression analyses to examine the impact of caregiver intuitive eating on change in %EBW at session 4 and EOT, when statistically adjusting for gender and whether or not the adolescent started treatment as an inpatient or outpatient. This analysis provides more information than a bivariate correlation, as it provides information regarding how much variance in change in %EBW over time can be attributed to baseline measures when taking adolescent gender and experience of inpatient treatment into account. Given that our sample size was too small to have enough power to detect significant effects with all the potential predictors, we focused on the overall score for caregiver IE, and the subscale score for caregiver “Eating for Physical Reasons.” We chose this subscale as this scale was the only significant predictor of weight gain in pounds or change in %EBW among all the IE subscales. (Additionally, no other baseline measures were significantly associated with any weight change variable at the bivariate correlation level).

We modeled 1. the impact of caregiver total IES scores on change in % EBW at session 4 when adjusting for gender and initial level of care; 2. The impact of the impact of caregiver total IES scores on change in % EBW at EOT when adjusting for gender and initial level of care; 3. The impact of the impact of caregiver Eating for Physical Reasons scores on change in % EBW at session 4 when adjusting for gender and initial level of care; and 4. The impact of the impact of caregiver Eating for Physical Reasons scores on change in % EBW at EOT when adjusting for gender and initial level of care. Gender and a dummy coded initial level of care variable (inpatient vs. outpatient) were entered in step 1 of the model. Caregiver IES scores (either total or Eating for Physical Reasons) were entered in the second step of the model (see Tables 4 and 5).

**Table 4:** Multiple regression analysis of gender, LOC, and caregiver total IES on change in %EBW at session 4 and EOT

	*R* (*R*^2^)	Change in %EBW session 4				*R* (*R*^2^)	Change in %EBW EOT			
		*R*^2 change^	*F*	*β*	*p*		*R*^2 change^	*F*	*β*	*p*
Step 1	0.295 (0.087)	0.09	2.05		0.142	0.170 (0.029)	0.03	0.547		0.572
Gender				− 0.30	0.054				− 0.10	0.548
Initial LOC				− 0.13	0.382				− 0.16	0.326
Step 2	0.381 (0.145)	0.06			0.099	0.347 (0.120)	0.09	3.834		0.058
Gender				− 0.33	0.031*				− 0.14	0.374
Initial LOC				− 0.12	0.41				− 0.17	0.315
Caregiver IES total				0.24	0.099				0.305	0.058

*LOC* level of care (inpatient or outpatient), *IES* Intuitive Eating Scale, *%EBW* percent change in expected body weight, *EOT* end of treatment

*p < 0.05

**Table 5:** Multiple regression analysis of gender, LOC, and caregiver EPR on change in %EBW at session 4 and EOT

	*R* (*R*^2^)	Change in %EBW session 4				*R* (*R*^2^)	Change in %EBW EOT			
		*R*^2 change^	*F*	*β*	*p*		*R*^2 change^	*F*	*β*	*p*
Step 1	0.295 (0.087)	0.04	2.047		0.142	0.170 (0.029)	0.03	0.567		0.572
Gender				− 0.30	0.054				− 0.10	0.548
Initial LOC				− 0.13	0.382				− 0.16	0.326
Step 2	0.399 (0.159)	0.07	3.260		0.064	0.360 (0.129)	0.10	4.27		0.046*
Gender				− 0.30	0.047				− 0.11	0.504
Initial LOC				− 0.07	0.642				− 0.11	0.505
Caregiver EPR				0.276	0.064				0.32	0.046

*LOC* level of care (inpatient or outpatient), *IES* Intuitive Eating Scale, *%EBW* percent change in expected body weight, *EOT* end of treatment

*p < 0.05

The first and second steps of the first model were not significant. This indicated that neither gender, initial level of care, nor caregiver total IES were statistically significantly associated with change in %EBW at session 4. In the second model, neither step was significant. Similarly, the first and second steps of the third model were not statistically significant. This indicated that neither gender, initial level of care, nor caregiver Eating for Physical Reasons were statistically significantly associated with change in %EBW at session 4. However, in the final model, caregiver Eating for Physical Reasons accounted for significant variance in change in %EBW at EOT when adjusting for gender and initial level of care (beta = 0.32, *p* < 0.046). Individual differences in caregiver scores on this subscale added an additional 10% of the variance to change in %EBW at EOT beyond that of gender and initial level of care. Notably, initial level of care did not contribute significant variance to change in %EBW in any of the models, suggesting that parental behavior during the outpatient phase of treatment had a greater impact on change in weight at EOT than initial treatment modality.

## Discussion

To our knowledge, this study is the first to explore caregiver and adolescent IE in a clinical sample of adolescents with AN or AAN. Additionally, it is the first study to examine the associations between caregiver and adolescent IE and weight gain in a FBT treatment-seeking sample. Several findings were consistent with previous literature, and several findings were novel. First, adolescent IE was negatively associated with measures of eating pathology and clinical impairment. This is consistent with previous literature in healthy adolescents yet notably the first known study in patients with AN or AAN at the start of FBT. Most notably, caregiver IE was negatively associated with weight and shape concerns on EDE-Q subscales and overall eating pathology and was positively associated with change in %EBW at EOT, as well as weight gain in pounds by session 4. This suggests that caregiver modeling of IE may impact body image concerns in their adolescents, even when they are ill with an ED. It may be that caregiver IE is associated with caregivers’ personal weight and shape concerns which are in turn modeled to their adolescent, consistent with literature on parent modeling of eating behavior in healthy youth [e.g., 17, 21].

Our finding that caregiver IE was associated with change in %EBW by FBT EOT and with weight gain by session 4 suggests that caregiver attitudes towards eating should be further explored in early FBT. On the IES-2, caregiver scores on the ERP subscale, or greater tendencies to eat based on hunger versus emotional cues, were positively associated with change in %EBW. In FBT, providers use the phrase “food is medicine” to emphasize the importance of caregivers reversing the effects of malnutrition by re-nourishing their adolescent. Caregivers are coached to provide their adolescent with enough nourishment and to do so on a regular schedule, as their adolescent may be unable to accurately evaluate hunger and satiety cues. Thus, in the context of this treatment model, caregivers who already endorse eating for physiological reasons may adapt to FBT more readily. Our finding that some caregiver attitudes towards eating were associated with weight gain is consistent with the limited literature on parental factors that may influence FBT. For example, parental self-efficacy regarding re-nourishment mediated weight restoration in an RCT of FBT [29]. This accumulating data suggests that caregiver attitudes towards eating and weight should be explored in larger studies of FBT. Attitudes towards eating are modifiable and it may be helpful for clinicians to assess these types of beliefs when initiating FBT. Finally, caregiver and adolescent scores on some IES-2 subscales were positively associated. This again suggests that caregiver modeling of IE may have some influence on their adolescent, even when the adolescent is ill with an ED.

Although not an aim of the current study, it is notable that change in %EBW by FBT session 4 was positively associated with change in %EBW by EOT. Secondary analyses of larger RCTs for FBT in adolescent AN have demonstrated this finding [23]. The fact that this finding was replicated in a sample of adolescents receiving FBT outside of the context of a controlled trial lends confidence to the hypothesis that weight gain by FBT session 4 is an important indicator of treatment success.

Finally, it is notable that there were no significant associations between eating pathology, clinical impairment, and change in %EBW at FBT session 4 and EOT. It is plausible that higher levels of clinical impairment may be associated with more difficulty with weight gain. However, we did not find this in our sample. This may be indicative of the efficacy of placing caregivers in charge of weight restoration and weight gain in phase I of FBT.

## Strengths and limits

This is the first known study to find associations between caregiver IE and weight change during treatment in a clinical sample of adolescents with AN or AAN. This study yielded several interesting findings, yet also had limitations. First, while the data are representative of adolescents with AN or AAN, the sample size is small. Interpretations should be made with caution given the chance of Type 1 error (false positive results). Future studies should work to replicate this study in a larger, and more diverse, sample of treatment-seeking adolescents. A larger sample would allow smaller effects to be detected that may not have been found in the current analyses. Further, a larger sample size may allow for comparisons of diagnostic groups, gender, race, and caregiver type. It is important to understand factors that influence treatment outcomes (weight gain) and how these effects may differ for certain adolescents.

Second, only baseline data was available for IES-2 scores. It is possible that adolescent and caregiver IE scores changed over the course of treatment, as caregivers learned strategies to feed their adolescent and as adolescents progressed in their recovery. Past research suggests that IE differs in individuals recovered from AN as compared to those with acute illness [6], so IE may change with weight restoration during FBT. Adolescents are likely better able to recognize and use IE after malnutrition is resolved. Related to this point, there is limited data on the construct validity of the IES-2 in adolescents with AN in acute illness stages. It is possible that their self-perceptions of their own eating behavior were skewed. For example, it is possible that adolescents with AN may perceive themselves as matching food choices to the needs of their body, and that this perception may change as they recover from the illness. In fact, Babbott et al. found that individuals with AN spectrum EDs had higher scores on the IES-2 than individuals with other ED diagnoses, with the highest scores on the EPR and BFCC subscales [42]. This finding is consistent with the scores obtained in our adolescent sample, in which the EPR and BFCC subscale scores were the highest. The fact that caregiver EPR was significantly positively associated with changes in weight (r values range from 0.28 to 0.35) while adolescent EPR was negatively associated with changes in weight (r values range from − 0.17 to 0.03, all non-significant) suggests that self-report patient IE has different meaning than self-report of IE in individuals without EDs, at least in the acutely ill phase prior to engaging in outpatient FBT.

Finally, as approximately two thirds of the sample were not deemed medically stable enough to start outpatient treatment, these adolescents were hospitalized, on average, for 2 weeks before starting outpatient FBT. Thus, these adolescents and their caregivers completed the baseline measures during the hospital admission. While all adolescents and caregivers completed the baseline assessments prior to starting FBT, the length of time prior to starting FBT from the time that they filled out questionnaires was varied. However, initial level of care (inpatient or outpatient) was not significantly associated with weight gain at the bivariate correlation level or in regression analyses.

Finally, our homogenous sample was a study limitation, with all but one adolescent identifying as White, non-Hispanic, and cisgender. Growing research illustrates the prevalence of EDs among racial/ethnic and gender diverse groups, as these illnesses do not discriminate [43]. Previous research suggests that in a sample of U.S. adults, White individuals were more likely to seek help for AN [44]. However, there may be systematic barriers and biases that further explain the lack of racial, ethnic, and gender diversity in this treatment-seeking sample. Additionally, adolescents who are marginalized for their sexual and gender identity have high rates of ED symptoms. In fact, transgender youth have higher rates of these symptoms than cisgender peers and may have unique risk and maintenance factors for disordered eating behavior [45]. It is crucial that future research explores these questions in a heterogenous sample of adolescents to understand how diversity impacts treatment outcomes in FBT.

## Future directions

Our study was largely exploratory and hypothesis generating, rather than hypothesis confirming. Future research should examine how IE changes throughout the course of treatment. Further, the relationship between changes in IE and changes in eating pathology should be examined to understand whether improvements in IE may be related to better treatment outcomes. Future research may benefit from understanding whether caregivers’ IE relates to their fidelity to FBT principles which in turn may benefit their adolescent. Another potential avenue for future research is to examine how addressing parent IE and other parent eating behavior in the first phase of treatment impacts their ability to engage in FBT. Future research may also examine the relationship between caregiver and adolescent IE later in treatment when the adolescent has more autonomy in their food choices.

## What is already known on this subject?

IE is associated with more adaptive eating and negatively correlated with disordered eating behaviors in healthy adolescents and adults, and in adults with EDs. Caregiver feeding and eating behaviors are also associated with their child’s eating behavior. This study aims to address a gap in the literature as to whether caregiver intuitive eating behavior impacts their adolescent’s progress in FBT for AN.

## What does this study add?

This study is the first to provide evidence that caregiver IE is positively associated with adolescent weight gain in FBT and is the first to provide evidence that caregiver IE is negatively related to adolescent ED symptoms. The data suggests that the relationship between caregiver IE and outcomes in FBT should be examined in a larger sample as a potential caregiver intervention point.

## Data Availability

The datasets generated during and/or analyzed during the current study are available from the corresponding author on reasonable request.
